# Optimal Strategies of Product Price, Quality, and Corporate Environmental Responsibility

**DOI:** 10.3390/ijerph16234704

**Published:** 2019-11-26

**Authors:** Wei Peng, Baogui Xin, Yekyung Kwon

**Affiliations:** 1 Nonlinear Science Center, College of Economics and Management, Shandong University of Science and Technology, Qingdao 266590, China; pengweisd@foxmail.com; 2 Division of Global Business Administration, Dongseo University, Busan 47011, Korea; ykkwon@gdsu.dongseo.ac.kr

**Keywords:** corporate social responsibility (CSR), corporate environmental responsibility (CER), government subsidy, social welfare, feedback equilibrium

## Abstract

With the awakening of environmental consciousness, more and more firms desire to go “green” by shifting their focus of corporate social responsibility (CSR) from charitable contributions to environmental actions called corporate environmental responsibility (CER). We develop a monopoly differential game to depict optimal corporate strategies of product price, quality, and CER. Using the Hamilton–Jacobi–Bellman (HJB) equation, we analyze optimal feedback equilibrium strategies for pricing and investing in both quality and CER with/without government subsidies. Numerical simulations show that government subsidy can improve CER and profit.

## 1. Introduction

As part and parcel of corporate social responsibility (CSR), corporate environmental responsibility (CER) complies with the rise of today’s environmental consciousness in environmental evolutions such as climate change. CER encompasses all the practices put in place by firms to reduce emissions, increase efficiency, and integrate sustainability into their daily operations. Employees, consumers, and stakeholders are placing a premium on working for, spending their money on, and standing by brands or companies that prioritize CER. Laudable green CER strategies can improve managerial altruism, consumer loyalty, corporation recommendations, brand sentiment, and cost-cutting efficiency. For that reason, more and more companies desire to go “green”. Therefore, it is meaningful for us to consider CER when we analyze firms’ decisions.

In recent years, more and more researchers also have paid much attention to CSR and CER [1,2,3,4,5,6]. Qin et al. [7] constructed a CER conceptual framework for researchers and proposed a conceptual model for policymakers. Suganthi [8] examined a general research framework considering CSR, green practice performance, and employees’ pro-environmental behavior. CSR can not only put enterprises into competitive disadvantage due to investment in CSR [9,10,11] but also help firms gain competitive advantage, because environmentally responsible behavior can obtain support from stakeholders (e.g., governments, suppliers, consumers, employees, and local communities) [12], expand their market share [9], reduce operational risk, and obtain long-term growth [13]. Some other researchers [14,15,16] have also indicated that CSR has a positive effect on corporate profits from different perspectives. Generally speaking, CSR helps corporations to gain better corporate goodwill [17,18,19], and better corporate goodwill helps corporations acquire more resources, and earn optimal profits. Examining the role of CER in CSR, Liu et al. [20] found that CER is positively associated with CSR to a significant degree. Dang et al. [21] insisted that CER is also a double-edged sword under different mediation effects, such as strategic similarity and organizational slack. Han, Yu, and Kim [3] uncovered that CER is a significant contributor to improving corporate goodwill and loyalty intentions. In the following, we will extend the Nerlove–Arrow model [22] to construct a monopoly differential game model by incorporating the effect of product quality, price, and CER on corporate goodwill to explore optimal corporate strategies.

The remainder of this paper is organized as follows. We review the relevant literature in Section 2. We propose a differential monopoly game model in Section 3. We analyze the equilibria without government subsidies in Section 4. We study the equilibria with government subsidies in Section 5. We validate the results by numerical simulations in Section 6. We discuss the results in Section 7. Finally, the paper concludes in Section 8.

## 2. Literature Review

Though there is no widely accepted definition of CER [7,8,23,24,25,26,27,28,29,30], for the sake of convenience, we support that CER is one of three facets of CSR, and focuses on pollution prevention and cleaner production. Furthermore, we regard the following terms as equivalent to CER: CSR in the environment, environmental CSR, environmental corporate responsibility. Like CSR, CER can impact the performance of micro-, small- and medium-sized enterprises from financial and innovative standpoints. As a kind of CSR, corporate contributions to charity may also have a long-term effect on a firm’s image and profits [31,32]. CER can facilitate firms to achieve support from external stakeholders, gain competitive advantages [33], reduce equity financing costs [34], affect investment efficiency for the long-term [35] and in green IT capital [36]. 

There is some literature about relationships between price, quality, and corporate responsibility (CR), as shown in Table 1. In this study, we analyze the relationship between price, quality, and CER by using the infinite-time differential game. Since the differential game will be used to analyze optimal corporate strategies of price, quality, and CER, some applications of the differential game are reviewed, as shown in Table 2. In this study, we investigate the feedback equilibria by setting corporate goodwill and CER knowledge accumulation as state variables, and setting pricing, investing in quality, and CER as control variables.

## 3. Model Formulation and Notation

**Figure 1:**
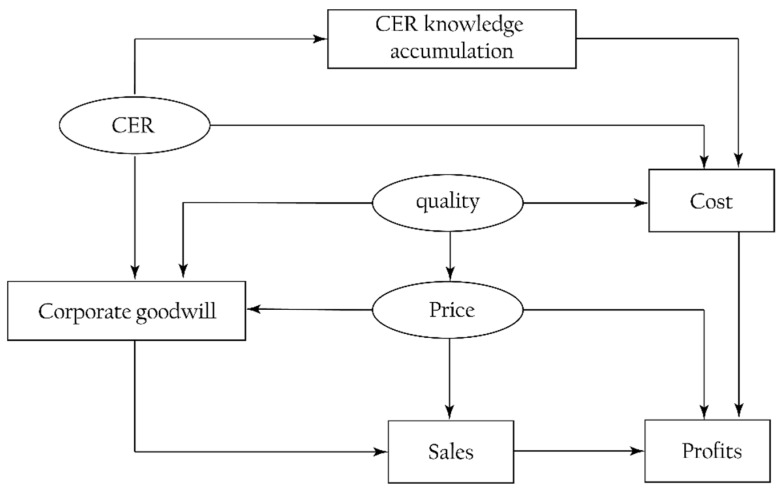
Block diagram of the proposed model.

As explained in Section 1 and Section 2, we consider an optimal dynamic problem over infinite time, in which a monopolist produces a single product and implements CER to promote corporate goodwill. General speaking, consumers are inclined to associate high quality and CER with high prices, where higher prices and CERs improve the corporate goodwill. Corporate goodwill directly affects sales. CER knowledge accumulation and investment in CER and quality all affect the cost. Besides, the classical supply–demand theory shows that (i) price is adversely related to sales, and (ii) the cost negatively affects the profits. We depict these relationships in the following block diagram, as shown in Figure 1.

Table 3 and Table 4 list the main notations used throughout the paper.

Incorporating the effects of price p(t), investment in quality z(t) and CSRI u(t) on corporate goodwill x(t), we extend the well-known Nerlove–Arrow model [22] to the following dynamic equation describing the time evolution of the corporate goodwill:

(1) $$\begin{array}{cc} \dot{x}(t)=k_{1}(p(t)-\bar{p})+k_{2}z(t)+k_{3}u(t)-\delta x(t), & x(0)=x_{0}. \end{array}$$

To formulate the demand problem in the monopolistic market, we extend the inverse demand function to the following demand function D(t), which depends jointly on the investment in quality z(t), price p(t), and corporate goodwill x(t):

(2) $$D(t)=a+a_{1}z(t)-a_{2}p(t)+a_{3}x(t).$$

According to [60,61], we employ the following linear marginal cost function of unitary production:

(3) $$C_{p}(z(t))=\eta z(t).$$

Borrowing from the thought of [62], we employ the following equation to measure the CSR knowledge accumulations:

(4) $$s(t)=s_{0}+\sigma \int_{0}^{t}u(h)dh,$$

which can be differentiated w.r.t. time t and gives

(5) $$\dot{s}(t)=\sigma u(t).$$

Inspired by [62,63], we consider the monopolist’s cost function of CSR as follows:

(6) $$C_{CER}\left(u(t),s(t)\right)=b_{1}u^{2}(t)-b_{2}\left(s(t)-s_{0}\right).$$

In this paper, we assume that all the demand can be satisfied, and there is no stock. We regard the demand function (2) as the product quantity under this circumstance. Then we can obtain the following monopolist’s instantaneous profits without government subsidies:

(7) $$\begin{array}{cc} \pi_{1}(t) & =\left(p(t)-C_{p}\left(z(t)\right)\right)D(t)-C_{CER}\left(u(t),s(t)\right)\\ & =\left(p(t)-\eta z(t)\right)\left(a+a_{1}z(t)-a_{2}p(t)+a_{3}x(t)\right)-b_{1}u^{2}(t)+b_{2}\left(s(t)-s_{0}\right). \end{array}$$

In the real world, a government tends to provide subsidies for firms that undertake CSR. In the following, we will explore the difference of monopolist’s optimal strategies between the case with and without government subsidies. For the sake of simplicity, we employ the following linear marginal subsidy function:

(8) $$G_{S}\left(u(t)\right)=b_{0}u(t).$$

Referring to Equation (7), we write the following instantaneous profits with government subsidies:

(9) $$\begin{array}{cc} \pi_{2}(t) & =\left(p(t)-C_{p}\left(z(t)\right)\right)D(t)-C_{CER}\left(u(t),s(t)\right)+G_{S}(u(t))\\ & =\left(p(t)-\eta z(t)\right)\left(a+a_{1}z(t)-a_{2}p(t)+a_{3}x(t)\right)+b_{0}u(t)-b_{1}u^{2}(t)+b_{2}\left(s(t)-s_{0}\right). \end{array}$$

To get the optimal combination of the product price, product quality, and CSRI to maximize its discounted infinite-horizon profit stream with/without government subsidies under the evolution of the corporate goodwill and CSR knowledge accumulations, we can depict it as the following differential game model:

(10) $$\begin{array}{cc} \underset{p(t),z(t),u(t)}{\max}\Pi =\int_{0}^{\infty }\;e^{-rt}\pi_{i}(t)dt, & i=1,2, \end{array}$$

(11) $$s.t.\{\begin{array}{l} \dot{s}(t)=\sigma u(t),\\ \dot{x}(t)=k_{1}(p(t)-\bar{p})+k_{2}z(t)+k_{3}u(t)-\delta x(t). \end{array}$$

where p(t), z(t), and u(t) are control variables; s(t) and x(t) are state variables.

## 4. The Case without Government Subsidy

In this section, we will perform the open-loop, closed-loop, feedback equilibrium analysis for the case without government subsidies. Moreover, the subscript F indicates the feedback equilibrium of variables. For simplicity, the time-dependence (t) of variables and state will be suppressed if no confusion arises.

### 4.1. The Open-/Closed-loop Equilibrium

We write the Hamiltonian function $H_{1}$ for the optimization model in Equations (10) and (11) without government subsidy as follows:

(12) $$\begin{array}{ccc} H_{1} & = & \left(p-\eta z\right)\left(a+a_{1}z-a_{2}p+a_{3}x\right)-b_{1}u^{2}+b_{2}\left(s-s_{0}\right)\\ & & +\lambda_{11}\sigma u+\lambda_{12}\left(k_{1}(p-\bar{p})+k_{2}z+k_{3}u-\delta x\right) \end{array}$$

where $\lambda_{11}$, $\lambda_{12}$ denote the dynamic adjoint variables related to their respective state equations $\dot{s}$ and $\dot{x}$ under the case without government subsidies.

From the Hamiltonian function $H_{1}$ in Equation (12), we get the first conditions for p,z, and u as follows:

(13) $$\frac{\partial H_{1}}{\partial p}=a+a_{1}z-2a_{2}p+a_{3}x+a_{2}z\eta +\lambda_{11}k_{1}=0$$

(14) $$\frac{\partial H_{1}}{\partial z}=\left(a_{2}p-a-a_{3}x\right)\eta +a_{1}\left(p-2z\eta \right)+\lambda_{11}k_{2}=0$$

(15) $$\frac{\partial H_{1}}{\partial u}=-2b_{1}u+\lambda_{11}k_{3}+\lambda_{12}\sigma =0$$

As mentioned in Section 3, where $a_{1}$, $a_{2}$, $b_{1}$, and η are positive parameters, the following sufficient optimality conditions for Equation (12) always hold:

$\frac{\partial^{2}H_{1}}{\partial p^{2}}=-2a_{2}<0$, $\frac{\partial^{2}H_{1}}{\partial z^{2}}=-2a_{1}\eta <0$, $\frac{\partial^{2}H_{1}}{\partial u^{2}}=-2b_{1}<0$,

$\frac{\partial^{2}H_{1}}{\partial p^{2}}\frac{\partial^{2}H_{1}}{\partial z^{2}}-{\left(\frac{\partial^{2}H_{1}}{\partial p\partial z}\right)}^{2}=-{\left(a_{1}-a_{2}\eta \right)}^{2}\leq 0$,

$\frac{\partial^{2}H_{1}}{\partial p^{2}}\frac{\partial^{2}H_{1}}{\partial u^{2}}-{\left(\frac{\partial^{2}H_{1}}{\partial p\partial u}\right)}^{2}=4a_{2}b_{1}>0$,

$\frac{\partial^{2}H_{1}}{\partial z^{2}}\frac{\partial^{2}H_{1}}{\partial u^{2}}-{\left(\frac{\partial^{2}H_{1}}{\partial z\partial u}\right)}^{2}=4a_{1}b_{1}\eta >0$.

As we know, $\frac{\partial^{2}H_{1}}{\partial p^{2}}\frac{\partial^{2}H_{1}}{\partial z^{2}}-{\left(\frac{\partial^{2}H_{1}}{\partial p\partial z}\right)}^{2}\leq 0$ denotes that the Hamiltonian function $H_{1}$ has no optimal solution. Therefore there is no open-/closed-loop equilibrium.

### 4.2. The Feedback Equilibrium

Several previous researchers [64] have proved that the feedback equilibrium policy fits the data better than the open-loop ones. Moreover, a feedback solution can better reflect the game dynamics over time. Therefore, in this section, we will perform a feedback equilibrium analysis for the differential game in (10) and (11) without government subsidies.

The Hamilton–Jacobi–Bellman (HJB) equation of the differential game in Equations (10) and (11) is:

(16) $$\begin{array}{cc} rV_{1} & =\underset{p,z,u}{\max}\left\{\pi_{1}+\dot{s}V_{1s}+\dot{x}V_{1x}\right\}\\ & =\underset{p,z,u}{\max}\left\{\begin{array}{l} \left(p-\eta z\right)\left(a+a_{1}z-a_{2}p+a_{3}x\right)-b_{1}u^{2}+b_{2}\left(s-s_{0}\right)\\ +\sigma uV_{1s}+\left(k_{1}(p-\bar{p})+k_{2}z+k_{3}u-\delta x\right)V_{1x} \end{array}\right\}, \end{array}$$

where $V_{1}=V_{1}\left(x,s\right)$ denotes the value function without government subsidies, $V_{1x}=\frac{\partial V_{1}}{\partial x}$, $V_{1s}=\frac{\partial V_{1}}{\partial s}$.

From Equation (16), we obtain the following first-order conditions for p,z, and u:

(17) $$\{\begin{array}{l} a+a_{1}z+a_{2}(z\eta -2p)+a_{3}x+k_{1}V_{1x}=0,\\ -a\eta +a_{1}\left(p-2z\eta \right)+a_{2}p\eta -a_{3}x\eta +k_{2}V_{1x}=0,\\ -2b_{1}u+\sigma V_{1s}+k_{3}V_{1x}=0. \end{array}$$

Solving Equation (17), we get the optimal feedback equilibrium for p, z, and u, denoted by $p_{F}^{*}$, $z_{F}^{*}$, $u_{F}^{*}$, which are shown in the following Proposition 1.

**Proposition** **1.** *Without government subsidies, the optimal feedback equilibrium for p, z, and u are given by*

(18) $$p_{F}^{*}=\frac{1}{{\left(a_{1}-a_{2}\eta \right)}^{2}}\left(a_{2}\eta \left(-k_{2}V_{1x}+\left(a+a_{3}x\right)\eta \right)-a_{1}\left(k_{2}V_{1x}+\left(a+2k_{1}V_{1x}+a_{3}x\right)\eta \right)\right)$$

(19) $$z_{F}^{*}=\frac{1}{{\left(a_{1}-a_{2}\eta \right)}^{2}}\left(\left(a-k_{1}V_{1x}+a_{3}x\right)\left(-a_{1}+a_{2}\eta \right)-2V_{1x}\left(a_{1}k_{1}+a_{2}k_{2}\right)\right)$$

(20) $$u_{F}^{*}=\frac{1}{2b_{1}}\left(\sigma V_{1s}+k_{3}V_{1x}\right)$$

**Proposition** **2.** *Without government subsidies, the value function $V_{1}\left(x,s\right)$, and the steady state of variables $p_{F}^{\infty }$, $z_{F}^{\infty }$, $u_{F}^{\infty }$, $s_{F}^{\infty }$, and $x_{F}^{\infty }$ satisfy the following equations*

(21) $$V_{1}\left(x,s\right)=n_{0}+n_{1}x+n_{2}x^{2}+\frac{b_{2}}{r}s$$

(22) $$p_{F}^{\infty }=\frac{a_{2}\eta \left(-k_{2}\left(n_{1}+n_{2}x_{F}^{\infty }\right)+\left(a+a_{3}x_{F}^{\infty }\right)\eta \right)-a_{1}\left(\left(n_{1}+n_{2}x_{F}^{\infty }\right)\left(k_{2}+2k_{1}\eta \right)+\left(a+a_{3}x_{F}^{\infty }\right)\eta \right)}{n_{04}{}^{2}}$$

(23) $$z_{F}^{\infty }=\frac{1}{n_{04}{}^{2}}\left(a_{2}\left(a\eta +a_{3}x_{F}^{\infty }\eta \right)-a_{1}\left(a+a_{3}x_{F}^{\infty }\right)-\left(n_{1}+n_{2}x_{F}^{\infty }\right)\left(a_{1}k_{1}+2a_{2}k_{2}+a_{2}k_{1}\eta \right)\right)$$

(24) $$u_{F}^{\infty }=\frac{1}{2b_{1}r}\left(k_{3}r\left(n_{1}+n_{2}x_{F}^{\infty }\right)+b_{2}\sigma \right)$$

(25) $$s_{F}^{\infty }=\frac{1}{2b_{1}r}\sigma \left(k_{3}r\left(n_{1}+n_{2}x_{F}^{\infty }\right)+b_{2}\sigma \right)t+C_{1}$$

(26) $$x_{F}^{\infty }=\frac{r\left(a_{1}{}^{2}\left(k_{3}{}^{2}n_{1}-2b_{1}k_{1}\bar{p}\right)-2m_{0}+a_{2}m_{1}\right)+b_{2}k_{3}n_{04}{}^{2}\sigma }{r\left(m_{2}+m_{3}+m_{4}\right)}+C_{2}e^{-\frac{m_{2}+m_{3}+m_{4}}{2b_{1}n_{04}{}^{2}}t}$$

*where:*

$$\{\begin{array}{l} n_{0}=\frac{1}{4b_{1}n_{04}{}^{2}r^{3}}\left(n_{01}+n_{02}+n_{03}-4ab_{1}n_{1}n_{04}n_{05}r^{2}\right),\\ n_{01}=a_{1}{}^{2}\left(k_{3}{}^{2}n_{1}{}^{2}r^{2}-4b_{1}r^{2}\left(k_{1}n_{1}\bar{p}+b_{2}s_{0}\right)+2b_{2}k_{3}n_{1}r\sigma +b_{2}{}^{2}\sigma^{2}\right),\\ n_{02}=-2a_{1}\left(2b_{1}r^{2}\left(n_{05}-2a_{2}b_{2}s_{0}\eta -2a_{2}k_{1}n_{1}\bar{p}\eta \right)+a_{2}\eta {\left(k_{3}n_{1}r+b_{2}\sigma \right)}^{2}\right),\\ n_{03}=a_{2}\left(-4b_{1}r^{2}\left(k_{2}n_{1}{}^{2}n_{05}+a_{2}\left(k_{1}n_{1}\bar{p}+b_{2}s_{0}\right)\eta^{2}\right)+a_{2}\eta^{2}{\left(k_{3}n_{1}r+b_{2}\sigma \right)}^{2}\right),\\ n_{04}=a_{1}-a_{2}\eta ,\\ n_{05}=k_{2}+k_{1}\eta \\ n_{1}=\frac{n_{2}n_{04}\left(2ab_{1}n_{05}r+n_{04}\left(2b_{1}k_{1}\bar{p}r-b_{2}k_{3}\sigma \right)\right)}{r\left(a_{1}{}^{2}\left(k_{3}{}^{2}n_{2}-2b_{1}\left(r+\delta \right)\right)-2a_{1}n_{11}-a_{2}n_{12}\right)},\\ n_{11}=a_{2}k_{3}{}^{2}n_{2}\eta +a_{3}b_{1}n_{05}+2b_{1}\left(k_{1}n_{2}n_{05}-a_{2}\left(r+\delta \right)\eta \right),\\ n_{12}=2b_{1}\left(2k_{2}{}^{2}n_{2}-k_{2}\left(a_{3}-2k_{1}n_{2}\right)\eta +\left(a_{2}\left(r+\delta \right)-a_{3}k_{1}\right)\eta^{2}\right)-a_{2}k_{3}{}^{2}n_{2}\eta^{2},\\ n_{2}=\frac{4b_{1}n_{04}\left(n_{04}\left(r+\delta \right)+a_{3}n_{05}\right)}{a_{1}{}^{2}k_{3}{}^{2}-2a_{1}\left(a_{2}k_{3}{}^{2}\eta +2b_{1}k_{1}n_{05}\right)-a_{2}\left(4b_{1}k_{2}n_{05}-a_{2}k_{3}{}^{2}\eta^{2}\right)},\\ m_{0}=ab_{1}n_{04}n_{05}+a_{1}\left(a_{2}k_{3}{}^{2}n_{1}\eta +2b_{1}k_{1}\left(n_{1}n_{05}-a_{2}\bar{p}\eta \right)\right),\\ m_{1}=a_{2}k_{3}{}^{2}n_{1}\eta^{2}-2b_{1}\left(2k_{2}n_{1}n_{05}+a_{2}k_{1}\bar{p}\eta^{2}\right),\\ m_{2}=a_{1}{}^{2}\left(2b_{1}\delta -k_{3}{}^{2}n_{2}\right),\\ m_{3}=2a_{1}\left(b_{1}k_{2}\left(a_{3}+2k_{1}n_{2}\right)+\left(a_{3}b_{1}k_{1}+2b_{1}k_{1}{}^{2}n_{2}+a_{2}k_{3}{}^{2}n_{2}-2a_{2}b_{1}\delta \right)\eta \right),\\ m_{4}=a_{2}\left(4b_{1}k_{2}{}^{2}n_{2}-2b_{1}k_{2}\left(a_{3}-2k_{1}n_{2}\right)\eta -\left(2a_{3}b_{1}k_{1}+a_{2}k_{3}{}^{2}n_{2}-2a_{2}b_{1}\delta \right)\eta^{2}\right). \end{array}$$

**Proof.**  Substituting the optimal feedback equilibrium in Equations (18)–(20) into the HJB Equation (16) yields:

(27) $$rV_{1}=\underset{p,z,u}{\max}\left\{\begin{array}{l} \frac{1}{4b_{1}}{\left(k_{3}V_{1x}+\sigma V_{1s}\right)}^{2}+\\ \frac{1}{n_{04}{}^{2}}\left(\begin{array}{l} a_{2}b_{2}\eta \left(s-s_{0}\right)\left(2a_{1}+a_{2}\eta \right)-V_{1x}\left(k_{1}\bar{p}+x\delta \right){\left(a_{1}\eta +a_{2}\right)}^{2}\\ +a_{2}n_{05}V_{1x}\left(a+a_{3}x\right)-a_{1}V_{1x}n_{05}\left(a-a_{3}x\right)+V_{1x}{}^{2}n_{05}\left(a_{1}k_{1}-a_{2}k_{2}\right) \end{array}\right) \end{array}\right\}$$

Differentiating the value function in Equation (21) with respect to s and x, respectively, gives

(28) $$V_{1s}=\frac{b_{2}}{r}$$

(29) $$V_{1x}=n_{1}+n_{2}x$$

Substituting Equations (21), (28) and (29) into (27), and equating the coefficients on both sides of Equation (27), we get $n_{0},n_{1}$ and $n_{2}$.

Substituting Equations (28) and (29) into (18)–(20), we can obtain the steady state of price $p_{F}^{\infty }$, investment in quality $z_{F}^{\infty }$, and CSRI $u_{F}^{\infty }$, as shown in Equations (22)–(24).

Substituting Equations (22)–(24) into (11), and solving the differential equations, it yields the steady state of CSR knowledge accumulations $s_{F}^{\infty }$, corporate goodwill $x_{F}^{\infty }$, as shown in Equations (25) and (26). □

## 5. The Case with Government Subsidy

To find out the equilibrium difference between the case with and without government subsidy, in this section, we will perform the open-loop, closed-loop, feedback equilibrium analysis for the case with government subsidy. Moreover, the subscript FS indicates the feedback equilibrium of variables.

### 5.1. The Open-/Closed-loop Equilibrium

The Hamiltonian function $H_{2}$ for the differential game in Equations (10) and (11) with government subsidies is:

(30) $$\begin{array}{ccc} H_{2} & = & \left(p-\eta z\right)\left(a+a_{1}z-a_{2}p+a_{3}x\right)+b_{0}u-b_{1}u^{2}+b_{2}\left(s-s_{0}\right)\\ & & +\lambda_{21}\sigma u+\lambda_{22}\left(k_{1}(p-\bar{p})+k_{2}z+k_{3}u-\delta x\right) \end{array}$$

where $\lambda_{21}$, $\lambda_{22}$ denote the dynamic adjoint variables related to their respective state equations $\dot{s}$ and $\dot{x}$ with government subsidies.

From the Hamiltonian function $H_{2}$ (30), we get the first conditions for p,z, and u as follows:

(31) $$\frac{\partial H_{2}}{\partial p}=a+a_{1}z-2a_{2}p+a_{3}x+a_{2}z\eta +\lambda_{21}k_{1}=0$$

(32) $$\frac{\partial H_{2}}{\partial z}=\left(a_{2}p-a-a_{3}x\right)\eta +a_{1}\left(p-2z\eta \right)+\lambda_{21}k_{2}=0$$

(33) $$\frac{\partial H_{2}}{\partial u}=b_{0}-2b_{1}u+\lambda_{21}k_{3}+\lambda_{22}\sigma =0$$

As mentioned in Section 3, $a_{1}$, $a_{2}$, $b_{1}$, and η are positive parameters, the following sufficient optimality conditions for Model (30) always hold:

$\frac{\partial^{2}H_{2}}{\partial p^{2}}=-2a_{2}<0$, $\frac{\partial^{2}H_{2}}{\partial z^{2}}=-2a_{1}\eta <0$, $\frac{\partial^{2}H_{2}}{\partial u^{2}}=-2b_{1}<0$,

$\frac{\partial^{2}H_{2}}{\partial p^{2}}\frac{\partial^{2}H_{2}}{\partial z^{2}}-{\left(\frac{\partial^{2}H_{2}}{\partial p\partial z}\right)}^{2}=-{\left(a_{1}-a_{2}\eta \right)}^{2}\leq 0$,

$\frac{\partial^{2}H_{2}}{\partial p^{2}}\frac{\partial^{2}H_{2}}{\partial u^{2}}-{\left(\frac{\partial^{2}H_{2}}{\partial p\partial u}\right)}^{2}=4a_{2}b_{1}>0$,

$\frac{\partial^{2}H_{2}}{\partial z^{2}}\frac{\partial^{2}H_{2}}{\partial u^{2}}-{\left(\frac{\partial^{2}H_{2}}{\partial z\partial u}\right)}^{2}=4a_{1}b_{1}\eta >0$.

As we know, $\frac{\partial^{2}H_{2}}{\partial p^{2}}\frac{\partial^{2}H_{2}}{\partial z^{2}}-{\left(\frac{\partial^{2}H_{2}}{\partial p\partial z}\right)}^{2}\leq 0$, which denotes that the Hamiltonian function $H_{2}$, has no optimal solution. Therefore, there is also no open-/closed-loop equilibrium with government subsidies.

### 5.2. The Feedback Equilibrium

In the following, we will perform a feedback equilibrium analysis for the differential game model with government subsidies.

The Hamilton–Jacobi–Bellman (HJB) equation of the differential game in Equations (10) and (11) is:

(34) $$\begin{array}{cc} rV_{2} & =\underset{p,z,u}{\max}\left\{\pi_{2}+\dot{s}V_{2s}+\dot{x}V_{2x}\right\}\\ & =\underset{p,z,u}{\max}\left\{\begin{array}{l} \left(p-z\eta \right)\left(a+a_{1}z-a_{2}p+a_{3}x\right)+b_{0}u-b_{1}u^{2}+b_{2}\left(s-s_{0}\right)\\ +\sigma uV_{2s}+\left(k_{1}(p-\bar{p})+k_{2}z+k_{3}u-x\delta \right)V_{2x} \end{array}\right\}, \end{array}$$

where $V_{2}=V_{2}\left(x,s\right)$ denotes the value function with government subsidy, $V_{2x}=\frac{\partial V_{2}}{\partial x}$, $V_{2s}=\frac{\partial V_{2}}{\partial s}$.

From the HJB Equation (34), we obtain the following first-order conditions for p, z, and u:

(35) $$\{\begin{array}{l} a+a_{1}z+a_{2}\left(z\eta -2p\right)+a_{3}x+k_{1}V_{2x}=0\\ -a\eta +a_{1}\left(p-2z\eta \right)+a_{2}p\eta -a_{3}x\eta +k_{2}V_{2x}=0\\ b_{0}-2b_{1}u+\sigma V_{2s}+k_{3}V_{2x}=0 \end{array}$$

Solving Equation (35), we get the optimal feedback equilibrium for p, z, and u, denoted by $p_{FS}^{*}$, $z_{FS}^{*}$, $u_{FS}^{*}$, which are shown in the following Proposition 3.

**Proposition** **3.** *With government subsidies, the optimal feedback equilibrium for p, z, and u are given by*

(36) $$p_{FS}^{*}=\frac{1}{n_{04}{}^{2}}\left(a_{2}\eta \left(\left(a+a_{3}x\right)\eta -k_{2}V_{2x}\right)-a_{1}\left(k_{2}V_{2x}+\left(a+2k_{1}V_{2x}+a_{3}x\right)\eta \right)\right)$$

(37) $$z_{FS}^{*}=\frac{1}{n_{04}{}^{2}}\left(n_{04}\left(k_{1}V_{2x}-a-a_{3}x\right)-2V_{2x}\left(a_{1}k_{1}+a_{2}k_{2}\right)\right)$$

(38) $$u_{FS}^{*}=\frac{1}{2b_{1}}\left(b_{0}+\sigma V_{2s}+k_{3}V_{2x}\right)$$

**Proposition** **4.** *With government subsidies, the value function $V_{2}\left(x,s\right)$, and the steady state of variables $p_{FS}^{\infty }$, $z_{FS}^{\infty }$, $u_{FS}^{\infty }$, $s_{FS}^{\infty }$, and $x_{FS}^{\infty }$ satisfy the following equations*

(39) $$V_{2}\left(x,s\right)=n_{3}+n_{4}x+n_{5}x^{2}+\frac{b_{2}}{r}s$$

(40) $$p_{FS}^{\infty }=\frac{1}{n_{04}{}^{2}}\left(\eta \left(a+a_{3}x_{FS}^{\infty }\right)\left(a_{1}+a_{2}\eta \right)+\left(n_{4}+n_{5}x_{FS}^{\infty }\right)\left(2a_{1}k_{1}\eta -a_{2}k_{2}\eta -a_{1}k_{2}\right)\right)$$

(41) $$z_{FS}^{\infty }=\frac{1}{n_{04}{}^{2}}\left(n_{04}\left(a+a_{3}x_{FS}^{\infty }\right)+\left(n_{4}+n_{5}x_{FS}^{\infty }\right)\left(a_{1}k_{1}+2a_{2}k_{2}+a_{2}k_{1}\eta \right)\right)$$

(42) $$u_{FS}^{\infty }=\frac{1}{2b_{1}r}\left(n_{34}+k_{3}n_{5}rx_{FS}^{\infty }\right)$$

(43) $$s_{FS}^{\infty }=\frac{1}{2b_{1}r}\sigma \left(n_{34}+k_{3}n_{5}rx_{FS}^{\infty }\right)t+C_{3}$$

(44) $$x_{FS}^{\infty }=\frac{m_{5}+r\left(m_{6}-m_{7}+m_{8}\right)}{r\left(a_{1}{}^{2}m_{9}+m_{10}+m_{11}\right)}+C_{4}e^{-\frac{a_{1}{}^{2}m_{9}+m_{10}+m_{11}}{2b_{1}n_{04}{}^{2}}t}$$

*where*

$$\{\begin{array}{l} n_{3}=\frac{1}{4b_{1}n_{04}{}^{2}r^{3}}\left(n_{31}-2a_{1}n_{32}+a_{2}n_{33}\right),\\ n_{31}=a_{1}{}^{2}\left(r^{2}\left(b_{0}{}^{2}+2b_{0}k_{3}n_{4}+k_{3}{}^{2}n_{4}{}^{2}-4b_{1}\left(k_{1}n_{4}\bar{p}+b_{2}s_{0}\right)\right)+b_{2}\sigma \left(2n_{34}-b_{2}\sigma \right)\right),\\ n_{32}=2b_{1}r^{2}\left(an_{4}n_{05}+k_{1}{}^{2}n_{4}{}^{2}\eta -2a_{2}b_{2}s_{0}\eta +k_{1}n_{4}\left(k_{2}n_{4}-2a_{2}\bar{p}\eta \right)\right)+a_{2}n_{34}{}^{2}\eta ,\\ n_{33}=a_{2}n_{34}{}^{2}\eta^{2}-4b_{1}r^{2}\left(k_{2}n_{4}\left(\left(k_{1}n_{4}-a\right)\eta +k_{2}n_{4}\right)+\left(k_{1}n_{4}\left(a_{2}\bar{p}-a\right)+a_{2}b_{2}s_{0}\right)\eta^{2}\right),\\ n_{34}=b_{0}r+k_{3}n_{4}r+b_{2}\sigma ,\\ n_{4}=\frac{1}{r\left(a_{1}{}^{2}n_{41}+n_{42}+n_{43}\right)}n_{5}n_{04}\left(-2ab_{1}n_{05}r+n_{04}\left(b_{0}k_{3}r+b_{2}k_{3}\sigma -2b_{1}k_{1}\bar{p}r\right)\right),\\ n_{41}=2b_{1}\left(r+\delta \right)-k_{3}{}^{2}n_{5},\\ n_{42}=2a_{1}\left(\left(a_{3}+2k_{1}n_{5}\right)b_{1}n_{05}-a_{2}n_{41}\eta \right),\\ n_{43}=a_{2}\left(4b_{1}k_{2}{}^{2}n_{5}-2b_{1}k_{2}\left(a_{3}-2k_{1}n_{5}\right)\eta +\left(a_{2}n_{41}-2a_{3}b_{1}k_{1}\right)\eta^{2}\right),\\ n_{5}=\frac{1}{a_{1}{}^{2}k_{3}{}^{2}+a_{2}k_{3}{}^{2}\eta \left(a_{2}\eta -2a_{1}\right)-4b_{1}n_{05}\left(a_{2}k_{2}+a_{1}k_{1}\right)}4b_{1}n_{04}\left(n_{04}\left(r+\delta \right)+a_{3}b_{1}n_{05}\right),\\ m_{5}=b_{2}k_{3}n_{04}{}^{2}\sigma -2ab_{1}n_{04}n_{05}r,\\ m_{6}=a_{1}{}^{2}\left(m_{12}-2b_{1}k_{1}\bar{p}\right),\\ m_{7}=2a_{1}\left(a_{2}m_{12}\eta +2b_{1}k_{1}\left(n_{4}n_{05}-a_{2}\bar{p}\eta \right)\right),\\ m_{8}=a_{2}\left(a_{2}m_{12}\eta^{2}-2b_{1}\left(2k_{2}n_{4}n_{05}+a_{2}k_{1}\bar{p}\eta^{2}\right)\right),\\ m_{9}=2b_{1}\delta -k_{3}{}^{2}n_{5},\\ m_{10}=2a_{1}\left(b_{1}k_{2}\left(a_{3}+2k_{1}n_{5}\right)+\left(a_{3}b_{1}k_{1}+2b_{1}k_{1}{}^{2}n_{5}-a_{2}m_{9}\right)\eta \right),\\ m_{11}=a_{2}\left(4b_{1}k_{2}{}^{2}n_{5}-2b_{1}k_{2}\left(a_{3}-2k_{1}n_{5}\right)\eta -\left(2a_{3}b_{1}k_{1}-a_{2}m_{9}\right)\eta^{2}\right),\\ m_{12}=k_{3}\left(b_{0}+k_{3}n_{4}\right). \end{array}$$

**Proof.**  Substituting the optimal feedback equilibrium in Equations (36)–(38) into the HJB Equation (34) yields

(45) $$rV_{2}=\underset{p,z,u}{\max}\left\{\begin{array}{l} \frac{1}{4b_{1}}{\left(k_{3}V_{2x}+\sigma V_{2s}\right)}^{2}+\\ \frac{1}{n_{04}{}^{2}}\left(\begin{array}{l} a_{2}b_{2}\eta \left(s-s_{0}\right)\left(2a_{1}+a_{2}\eta \right)-V_{2x}\left(k_{1}\bar{p}+x\delta \right){\left(a_{1}\eta +a_{2}\right)}^{2}\\ +a_{2}V_{2x}\left(a+a_{3}x\right)\left(k_{1}+k_{2}\eta \right)-a_{1}V_{2x}n_{05}\left(a-a_{3}x\right)\\ +V_{2x}{}^{2}n_{05}\left(a_{1}k_{1}-a_{2}k_{2}\right) \end{array}\right) \end{array}\right\}$$

Differentiating the value function in Equation (39) with respect to s and x, respectively, gives

(46) $$V_{2s}=\frac{b_{2}}{r}$$

(47) $$V_{2x}=n_{4}+n_{5}x$$

Substituting Equations (39), (46), and (47) into (45), and equating the coefficients on both sides of Equation (45), we get $n_{3},n_{4}$ and $n_{5}$.

Substituting Equations (46) and (47) into (36)–(38), we can obtain the steady state of control variables $p_{FS}^{\infty }$, $z_{FS}^{\infty }$, and $u_{FS}^{\infty }$, as shown in Equations (40)–(42).

Substituting Equations (40)–(42) into (11), and solving the differential equations, it yields the steady state of state variables $s_{FS}^{\infty }$, and $x_{FS}^{\infty }$, as shown in Equations (43) and (44). □

## 6. Simulation

For the sake of convenience, we initialize parameters for the proposed model as follows.

$\bar{p}=150$, $x_{0}=2$, $s_{0}=5$, $k_{1}=0.01$, $k_{2}=1.8$, $k_{3}=0.5$, δ=0.01, $b_{0}=0.2$, $b_{1}=4$, $b_{2}=3$, a=80, $a_{1}=2.6$, $a_{2}=2$, $a_{3}=2.2$, σ=2, r=0.1, η=3.3. Optimal solutions with/without government subsidies are presented in the following.

(i) The case without government subsidies:

$p_{F}\left(t\right)=7.701919+0.398208e^{-1.010689t}$,

$z_{F}\left(t\right)=5.030651+0.917139e^{-1.010689t}$,

$u_{F}\left(t\right)=0.379791-0.030029e^{-1.010689t}$,

$s_{F}\left(t\right)=0.005942e^{-1.010689t}+0.075958t+4.994058$,

$x_{F}\left(t\right)=4.591009-2.591009e^{-1.010689t}$,

$V_{1}\left(t\right)=11.775529+2.489789e^{-2.021379t}-4.630587e^{-1.010689t}+2.278744t$.

(ii) The case with government subsidies:

$p_{FS}\left(t\right)=7.695105+0.636307e^{-1.010689t}$,

$z_{FS}\left(t\right)=5.026388+0.912762e^{-1.010689t}$,

$u_{FS}\left(t\right)=0.404778-0.029886e^{-1.010689t}$,

$s_{FS}\left(t\right)=0.005914e^{-1.010689t}+0.080956t+4.994086$,

$x_{FS}\left(t\right)=4.578642-2.578642e^{-1.010689t}$,

$V_{2}\left(t\right)=13.322593+2.466076e^{-2.021379t}-4.595591e^{-1.010689t}+2.428666t$.

In the following, t varies from 0 to 10 with an increment of 1 in all plots.

### 6.1. The Optimal Price Levels

**Figure 2:**
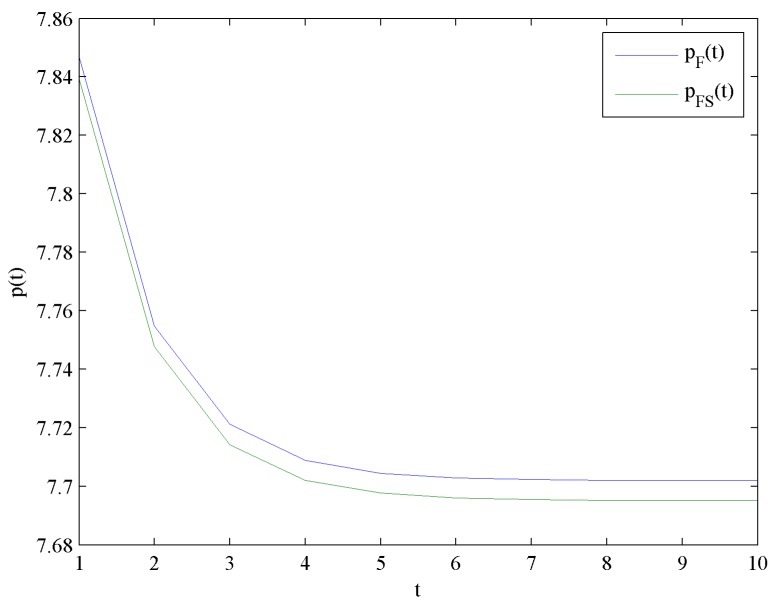
Evolutions of the optimal price levels.

Figure 2 presents the evolution trends of the optimal price levels $p_{F}$ and $p_{FS}$ by increasing time t. Figure 2 illustrates that $p_{F}$ and $p_{FS}$ decrease at first, and eventually reach steady levels $p_{F}^{\infty }=7.7019$ and $p_{FS}^{\infty }=7.6951$, respectively.

**Figure 3:**
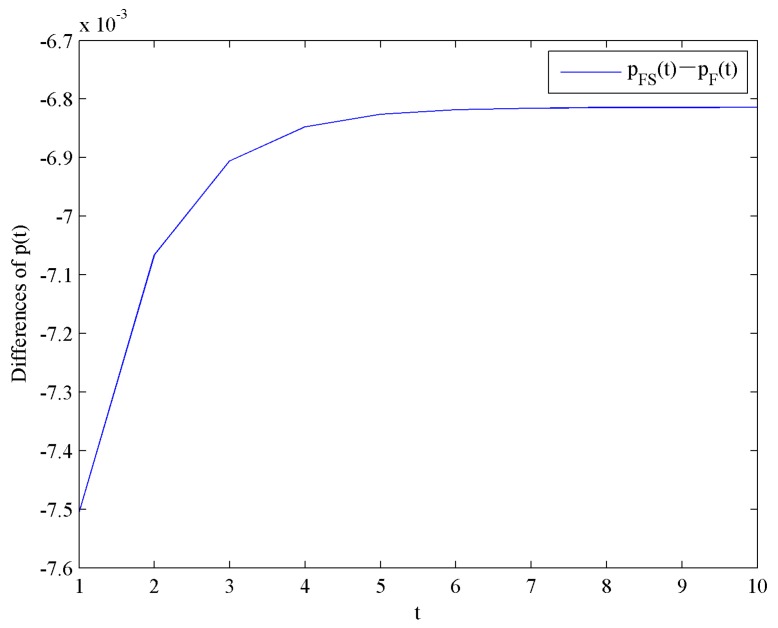
Difference in the optimal price levels.

This result shows that the optimal price level with government subsidies is lower than that without government subsidy. Moreover, the effect of government subsidy on the optimal price levels is shown in Figure 3.

### 6.2. The Optimal Investment Levels in Quality

**Figure 4:**
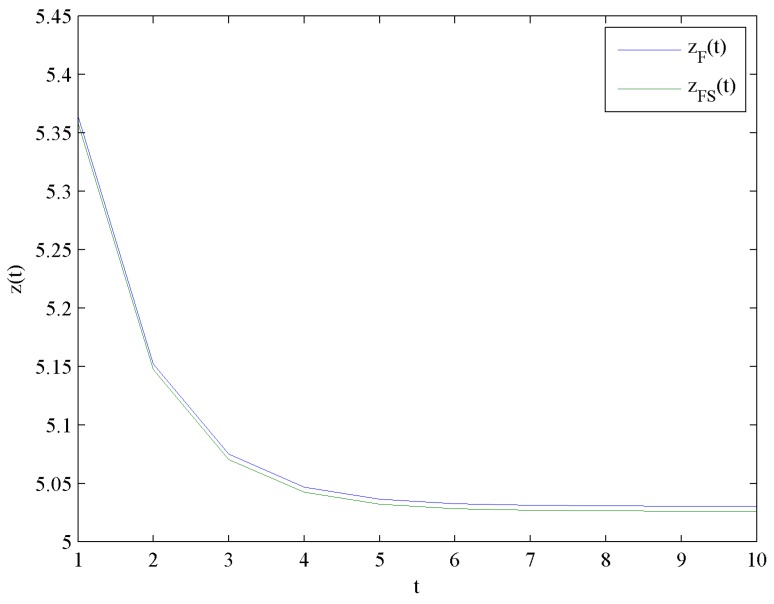
Evolutions of the optimal investment levels in quality.

Figure 4 illustrates the evolution trends of the optimal investment levels in quality $z_{F}$ and $z_{FS}$ by increasing time t. Figure 4 presents that $z_{F}$ and $z_{FS}$ decrease rapidly at the beginning, and eventually reach steady levels $z_{F}^{\infty }=5.0307$ and $z_{FS}^{\infty }=5.0264$, respectively.

**Figure 5:**
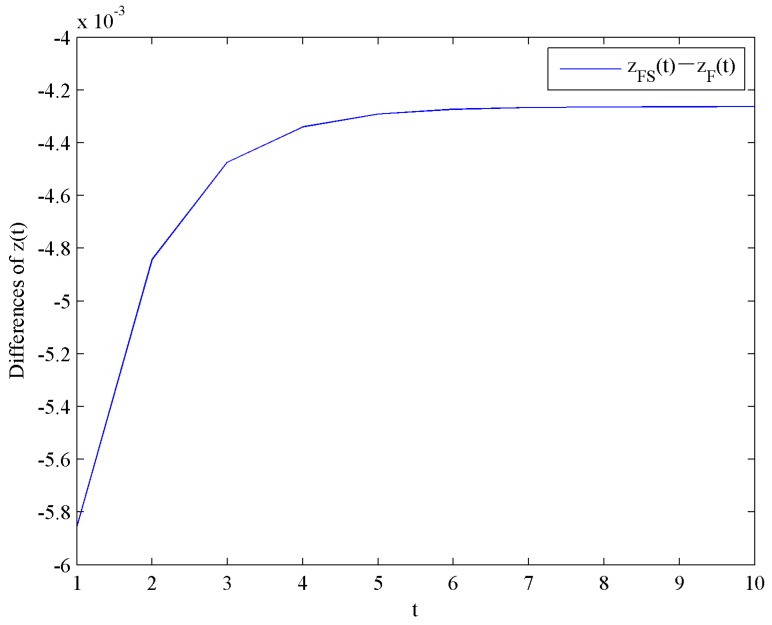
Difference of the optimal investment levels in quality.

This result shows that the optimal investment level in quality with government subsidies is lower than the case without government subsidies. Moreover, the effect of government subsidies on the optimal investment levels in quality is shown in Figure 5.

### 6.3. The Optimal Investment Levels in CER

**Figure 6:**
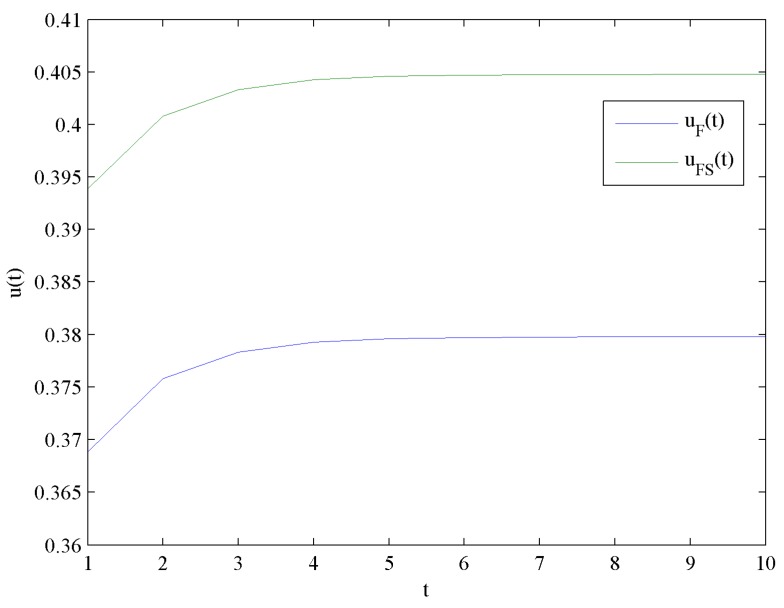
Evolutions of the optimal investment levels in CER.

Figure 6 presents the evolution trends of the optimal investment levels in CER $u_{F}$ and $u_{FS}$ by increasing time t. Figure 6 shows that $u_{F}$ and $u_{FS}$ increase at first, and eventually reach steady levels $u_{F}^{\infty }=0.3798$ and $u_{FS}^{\infty }=0.4048$, respectively.

**Figure 7:**
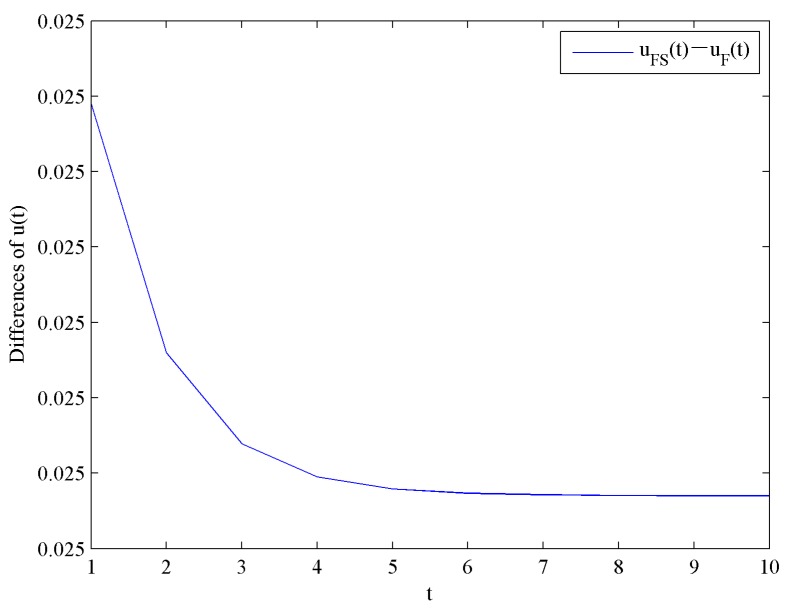
Difference of the optimal investment levels in CER.

Obviously, $u_{FS}^{\infty }>u_{F}^{\infty }$ holds, which means the optimal investment level in CER with government subsidies is higher than that without government subsidies. Moreover, the impact of government subsidies on the optimal investment levels in CER is shown in Figure 7.

### 6.4. The Optimal CER Knowledge Accumulations Levels

**Figure 8:**
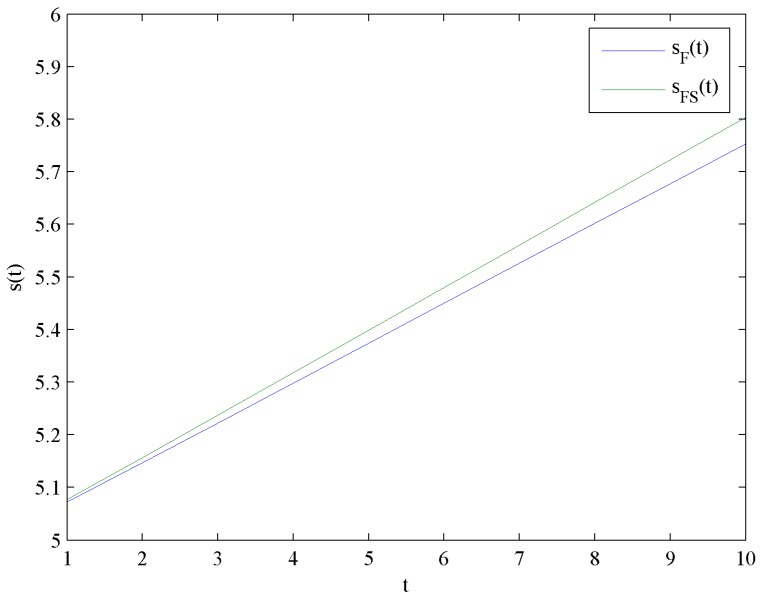
Evolutions of the optimal CER knowledge accumulation levels.

**Figure 9:**
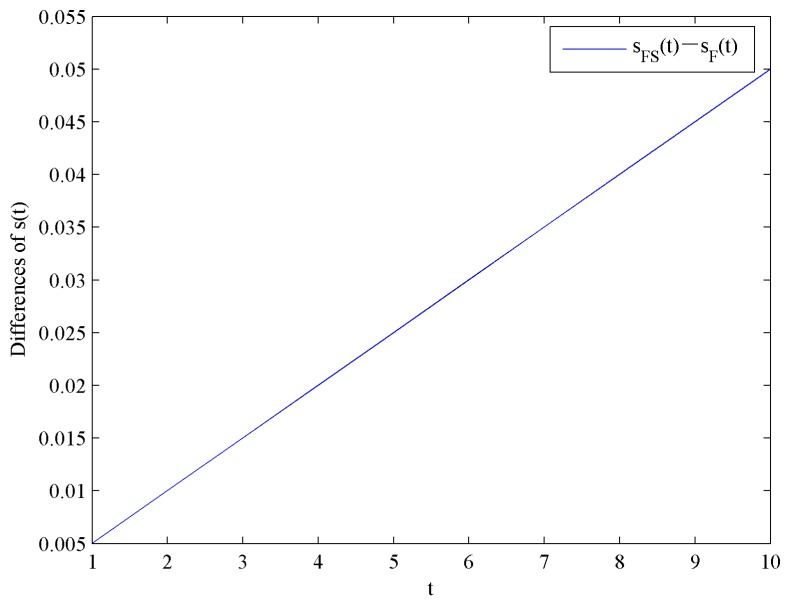
Difference of the optimal CER knowledge accumulation levels.

Figure 8 shows the evolution trends of the CER knowledge accumulations levels $s_{F}$ and $s_{FS}$ by increasing time t. Figure 8 illustrates that $s_{F}$ and $s_{FS}$ are in linear growth because the CER knowledge accumulations function in Equation (4) is linear. Moreover, the impact of government subsidies on the CER knowledge accumulations levels is shown in Figure 9.

### 6.5. The Optimal Corporate Goodwill Levels

**Figure 10:**
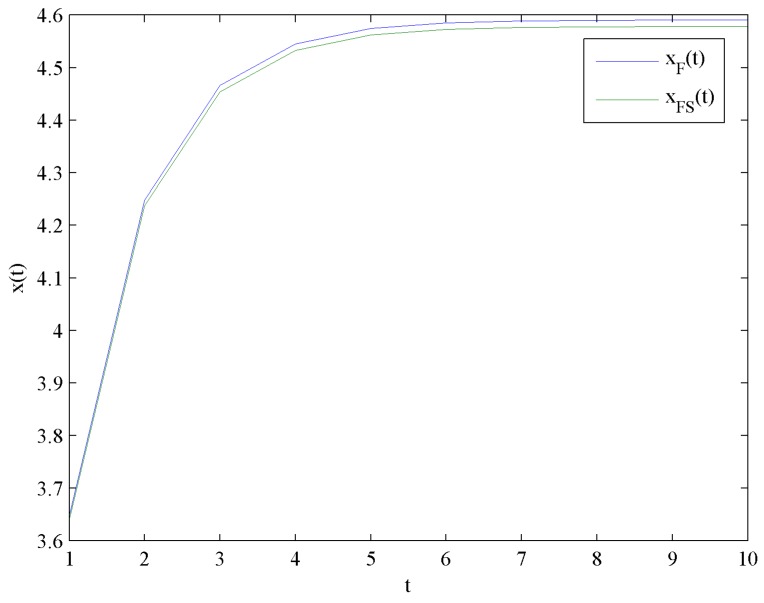
Evolution of the optimal corporate goodwill levels.

Figure 10 shows the evolution trends of the optimal corporate goodwill levels $x_{F}$ and $x_{FS}$ by increasing time t. Figure 10 illustrates that $x_{F}$ and $x_{FS}$ increase rapidly at the beginning, and eventually reach steady levels $x_{F}^{\infty }=4.5910$ and $x_{FS}^{\infty }=4.5786$, respectively.

**Figure 11:**
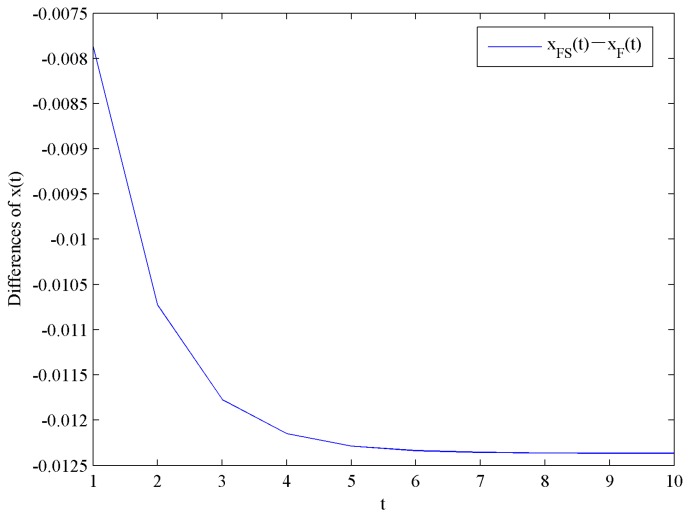
Difference in the optimal corporate goodwill levels.

This result shows that the optimal corporate goodwill level with government subsidies is lower than that without government subsidies. Moreover, the impact of government subsidies on the optimal corporate goodwill levels is shown in Figure 11.

### 6.6. The Optimal Value Functions

**Figure 12:**
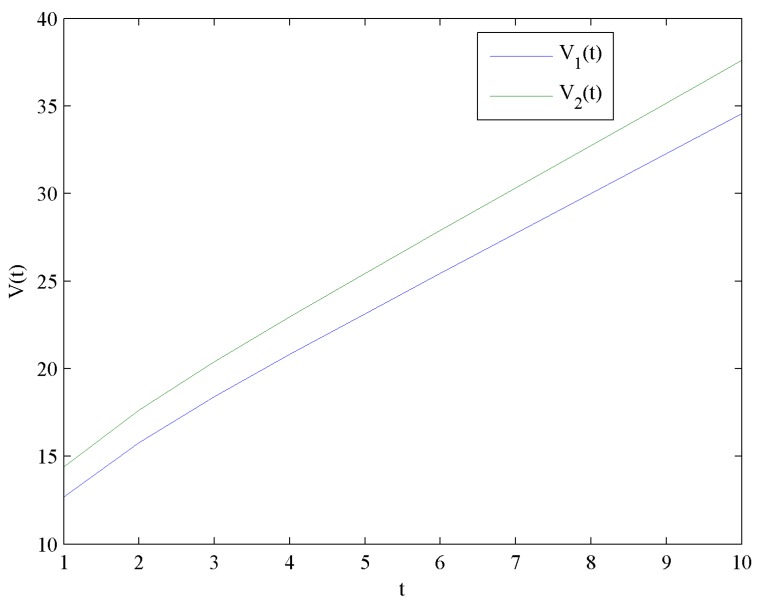
Evolution of the optimal value functions.

**Figure 13:**
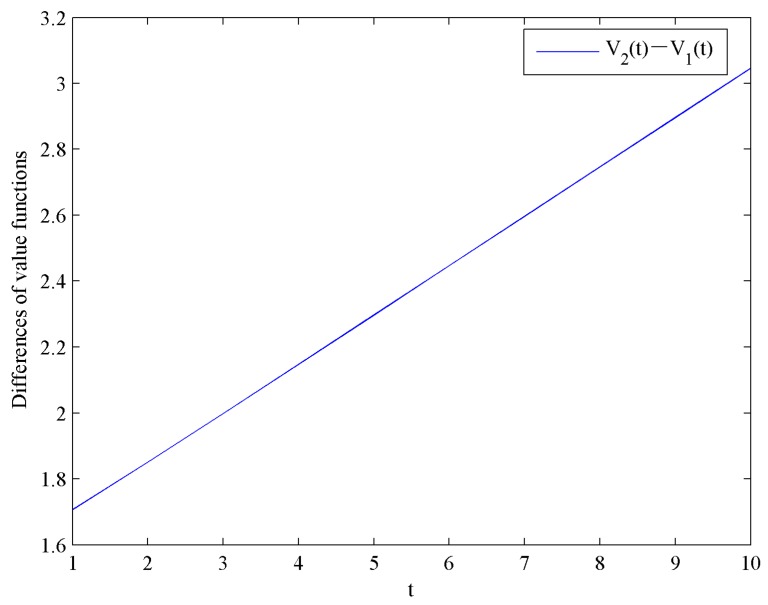
Difference of the optimal value functions.

Figure 12 shows the evolution trends of the value functions $V_{1}$ and $V_{2}$ by increasing time t. Figure 12 illustrates that $V_{1}$ and $V_{2}$ are continuously increasing. Moreover, the impact of government subsidies on value functions is shown in Figure 13.

### 6.7. The Effect of Control Variables on Value Functions

The value function reflects the firm’s profits. In this subsection, we simulate the effects of three control variables on value functions as follows.

**Figure 14:**
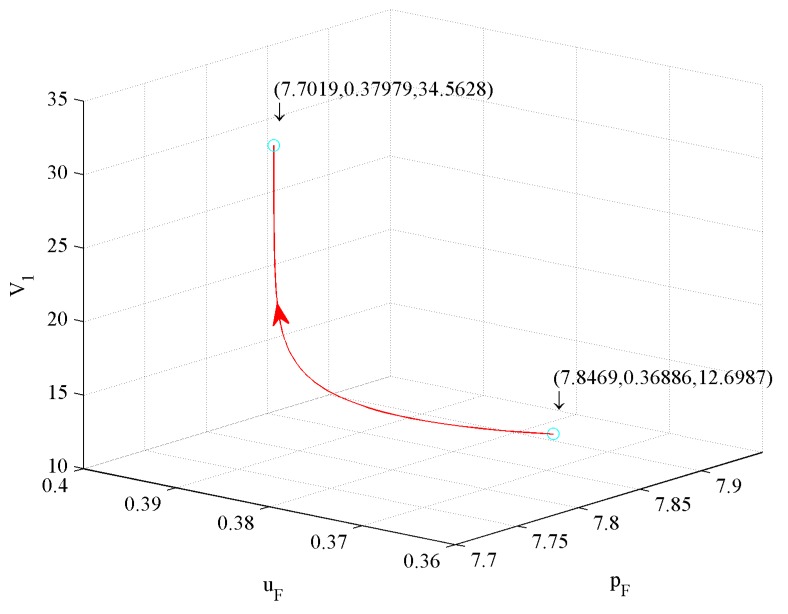
The effect of price and investment in CER on $V_{1}$.

Figure 14 shows the impact of price and investment in CER on value function $V_{1}$. The figure illustrates that a 1.85% price decrease and a 2.96% investment in CER increase drive a 172.18% $V_{1}$ increase.

**Figure 15:**
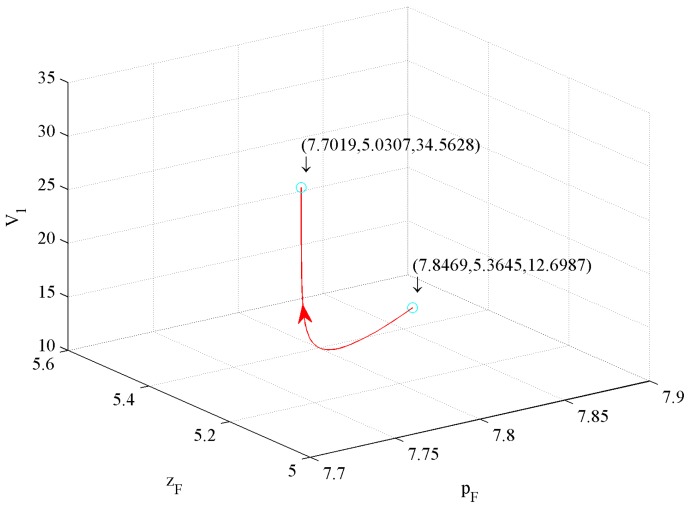
The effect of price and investment in quality on $V_{1}$.

Figure 15 shows the impact of price and investment in quality on value function $V_{1}$. The figure illustrates that a 1.85% price decrease and a 6.22% investment in quality decrease drive a 172.18% $V_{1}$ increase.

**Figure 16:**
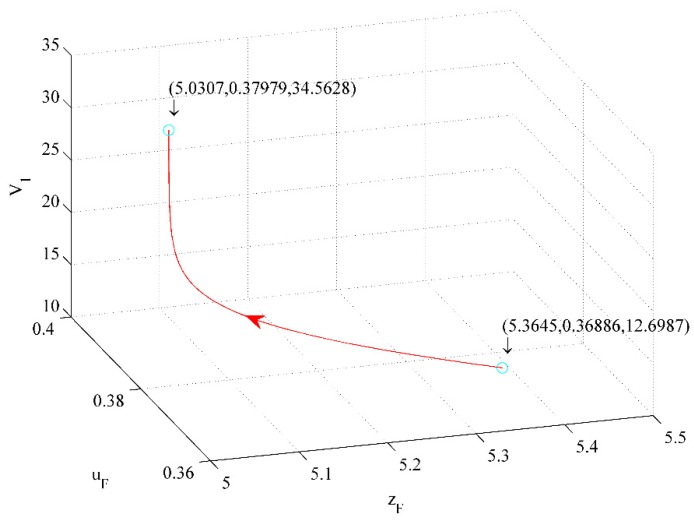
The effect of investment in quality and in CER on $V_{1}$.

Figure 16 shows the effect of investment quality and in CER on value function $V_{1}$. The figure illustrates that a 6.22% investment in quality decrease and a 2.96% investment in CER increase drive a 172.18% $V_{1}$ increase.

From Figure 14, Figure 15, and Figure 16, we conclude that the first influence factor on $V_{1}$ is price, the second one is the investment in CER, and the third one is the investment in quality.

**Figure 17:**
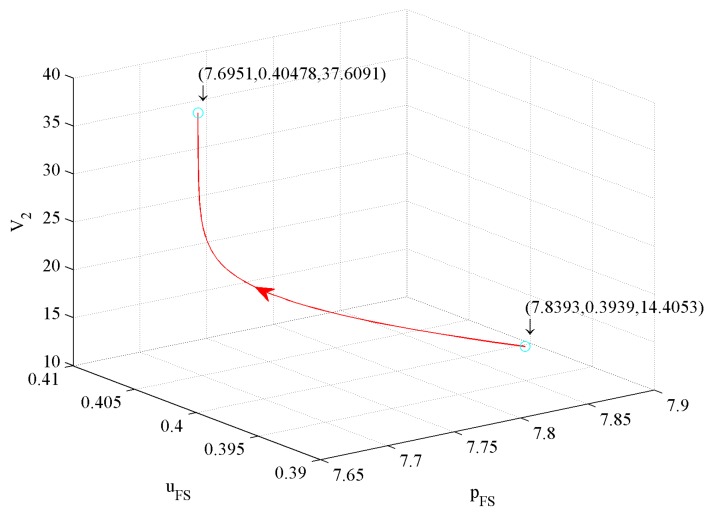
The effect of price and investment in CER on $V_{2}$.

Figure 17 shows the influence of price and investment in CER on the value function $V_{2}$. The figure illustrates that a 1.84% price decrease and a 2.76% investment in CER increase drive a 161.08% $V_{2}$ increase.

**Figure 18:**
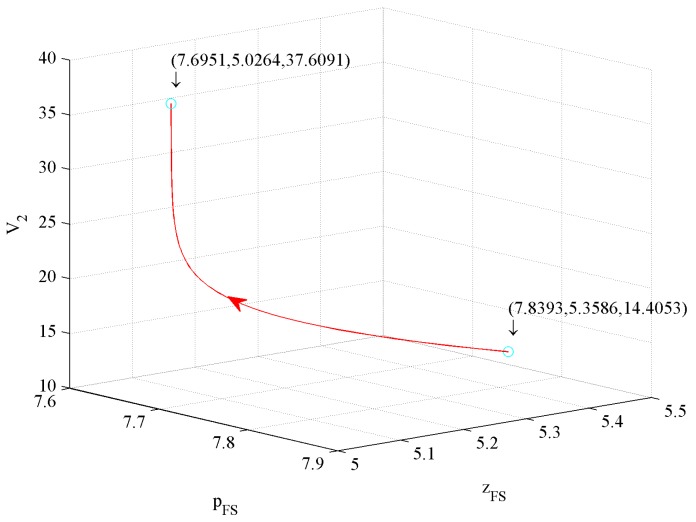
The effect of price and investment in quality on $V_{2}$.

Figure 18 shows the influence of price and investment in quality on value function $V_{2}$. The figure illustrates that a 1.84% price decrease and a 6.2% investment in quality decrease drive a 161.08% $V_{2}$ increase.

**Figure 19:**
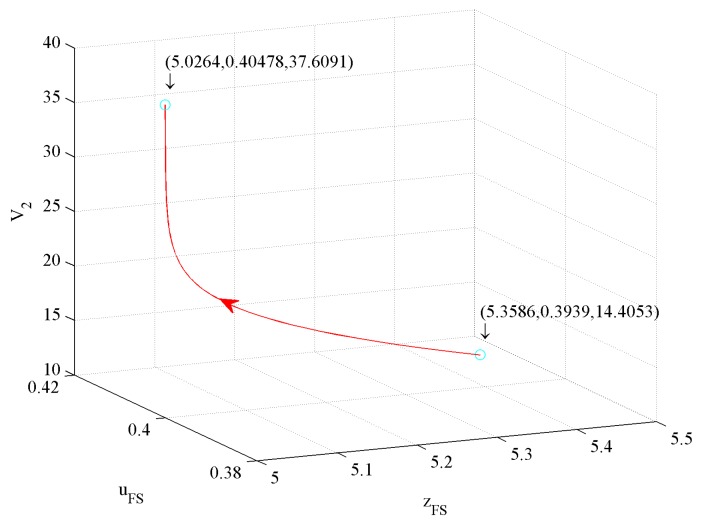
The effect of investment in quality and in CER on $V_{2}$.

Figure 19 shows the influence of investment in quality and CER on value function $V_{2}$. The figure illustrates that a 6.2% investment in quality decrease and a 2.76% investment in CER increase drive a 161.08% $V_{2}$ increase.

According to Figure 17, Figure 18 and Figure 19, we conclude that the first influence factor on $V_{2}$ is also price, the second one is also the investment in CER, and the third one is also the investment in quality.

To sum up, whether or not to consider government subsidies, the first influence factor on profit is price, the second one is the investment in CER, and the third one is the investment in quality. The profit with government subsidies is higher than that without government subsidies. However, the growth rate of profit with government subsidies is lower than that without government subsidies.

## 7. Discussions

Environmentally responsible firms tend to gain better corporate goodwill, while better corporate goodwill helps the enterprise achieve competitive advantages. Since CER is a spontaneous organization behavior, its actual effect is limited. Therefore, government involvement in firms’ CER practice is of great significance. Despite some researchers emphasizing that CER is vital for firms and governments, literature exploring how government subsidies affect firms’ optimal strategies when considering the impacts of price, quality, and CER on corporate goodwill, is scarce. To bridge this literature gap, we developed the monopoly differential game mentioned above to depict a joint optimization of pricing and investing in quality and CER with/without government subsidies. Results reveal that:

(1) Government subsidies have adverse effects on the optimal price, investment in quality, and corporate goodwill levels, and positively affect the optimal investment in CER, CER knowledge accumulations levels, and value functions.

(2) Considering government subsidies, the monopolist would increase the investment in CER. Comparing Equations (24) and (42), we find the investment increase in CER is only a part of government subsidies, which follows the profit-maximizing hypothesis.

(3) Whether or not to consider government subsidies, the first influence factor on profits is the price, the second one is the investment in CER, and the third one is the investment in quality. The profit with government subsidies is higher than that without government subsidies. The growth rate of profit with government subsidies is lower than that without government subsidies.

In this paper, we reveal the relationship between product price, quality, and CER in a monopoly market with/without government subsidies. Our results can guide enterprises in optimizing their overall decisions of product pricing, quality improvement, and investment in corporate environmental responsibility. It can guide enterprises to make rational pricing, continuously improve product quality, and consistently perform CER, which can increase social welfare. Our results also provide theoretical support for the government to regulate CER, supervise product quality, regulate pricing, and improve social welfare by using government subsidies.

## 8. Conclusions

In this paper, we construct a differential game over infinite time, in which a monopolist produces a single product and implements the investment in CER. We then explore an environmentally responsible monopolist’s feedback equilibrium strategies with/without government subsidies. Results show that government subsidies effect a monopolist’s optimal strategies. 

The following extensions are of interest for future research:

(1) The output of the proposed game is entirely determined by the parameter values and the initial conditions. However, the real world is disturbed by stochasticity. For further development, stochastic models that possess some inherent randomness can be considered, such as a stochastic differential game [65].

(2) We leveraged the linear CSR knowledge accumulations function, which can be further improved to a nonlinear function.

## Figures and Tables

**Table 1:** Main relationships between profit, price, quality, and CR.

Study	Main Relationship	Method	Reference
De Giovanni and Zaccour (2019)	Quality and product price	Two-stage model	[37]
Li et al. (2019)	Price and quality strategies	Tobit regression and ordinary least square models	[38]
Voros (2019)	Price and quality	Finite-time differential game	[39]
Zhao and Zhang (2019)	Price and quality	Dynamic programming model	[40]
Hosseini-Motlagh et al. (2019)	Price, sustainability level, and CSR	Stackelberg game	[41]
Khosroshahi et al. (2019)	Price, transparency, and CSR	Stackelberg game	[42]
Jeong and Yoon (2014)	Quality and CSR image	Empirical and causal approaches	[43]
Gatti, Caruana, and Snehota (2012)	CSR and perceived quality	Structural equation model	[44]
Nie, Wang, and Meng (2019)	CER and profit	Static game	[45]
Wong et al. (2018)	CER and income	Content analysis approach	[46]
Jiang, Xue and Xue (2018)	CER and performance	Multi-variables regression	[47]

**Table 2:** Differential games with more state or control variables.

Study	State Variables	Control Variables	Solution Type	Reference
Lin and Wang (2019)	Accumulation of sharing knowledge	Effort level of knowledge sharing, degree of incentive	Feedback	[48]
Jiang et al. (2019)	Pollutant stock	Emission capacity, pollution governance investment, eco-compensation ratio	Feedback	[49]
Xin and Sun (2018)	Product prices, water right prices	Production planning, water saving	Open-loop, closed-loop, feedback	[50]
Yang and Xu (2019)	Carbon stock, inventory level	Production output, product flow, product transaction, resource investment, carbon emission, carbon permit	Numerical	[51]
Lu, Zhang, and Tang (2019)	Corporation goodwill	Advertising effort, retail margin, wholesale price, profit rate of cost	Feedback	[52]
Wu (2018, 2019)	Network effect, innovation level	Price	Feedback	[53,54]
Xin, Peng, and Sun (2019)	Pollutant stock level	Production output, abatement effort	Feedback	[55]
Esfahani (2019)	Product price	Production output	Open-loop, closed-loop	[56]
Lu et al. (2019)	Product price	Order quantity, advertising effort, wholesale price	Feedback	[57]
Kicsiny and Varga (2019)	Water resource volume, payoff	consumption flow rate	Numerical	[58]
Chan, Zhou, and Wong (2019)	Cumulative profit	New production output	Numerical	[59]

**Table 3:** Variables and descriptions.

Variables	Description
p(t)	The product price at time t
u(t)	Investment in CER at time t
x(t)	The corporate goodwill at time t
s(t)	CER knowledge accumulation from time 0 to t
z(t)	Investment in product quality at time t
$C_{p}\left(z(t)\right)$	The marginal cost of production
$C_{CER}\left(u(t),s(t)\right)$	The cost of CER at time t
D(t)	The demand function at time t
$G_{S}\left(u(t)\right)$	The marginal government subsidy function at time t
$\pi_{i}(t)$	The net profit rate with at time t, i=1,2 denotes without and with government subsidy, respectively.

**Table 4:** Parameters and descriptions.

Parameters	Description
$\bar{p}$	The expected price for the brand with current corporate goodwill, $\bar{p}>0$
$x_{0}$	The initial level of corporate goodwill, $x_{0}\geq 0$
$s_{0}$	The initial CER knowledge accumulations, $s_{0}>0$
$k_{1}$	The price effect on the corporate goodwill, $k_{1}>0$
$k_{2}$	The effect of CER investment on the corporate goodwill, $k_{2}>0$
$k_{3}$	The effect of quality investment on the corporate goodwill, $k_{3}>0$
δ	The depreciation rate of the corporate goodwill, δ>0
$b_{0}$	The rate of government subsidy, $b_{0}\geq 0$
$b_{1}$	The effect of CER investment on CER cost, $b_{1}>0$
$b_{2}$	The learning rate of CER, $b_{2}>0$
a	The demand intercept, a>0
$a_{1}$	The effect of quality investment on demand, $a_{1}>0$
$a_{2}$	The price effect on demand, $a_{2}>0$
$a_{3}$	The corporate goodwill effect on demand, $a_{3}>0$
σ	The effect of CER investment on the knowledge accumulations, σ>0
r	The discount rate, r>0
$\lambda_{11}$, $\lambda_{12}$, $\lambda_{21}$, $\lambda_{22}$	Dynamic adjoint variables
η	The effect of quality investment on the margin production cost, η>0
$C_{1}$, $C_{1}$, $C_{1}$, $C_{1}$	Constants
